# Prediction of balance function for stroke based on EEG and fNIRS features during ankle dorsiflexion

**DOI:** 10.3389/fnins.2022.968928

**Published:** 2022-08-18

**Authors:** Jun Liang, Yanxin Song, Abdelkader Nasreddine Belkacem, Fengmin Li, Shizhong Liu, Xiaona Chen, Xinrui Wang, Yueyun Wang, Chunxiao Wan

**Affiliations:** ^1^Department of Rehabilitation, Tianjin Medical University General Hospital, Tianjin, China; ^2^Laboratory of Neural Engineering and Rehabilitation, Department of Biomedical Engineering, College of Precision Instruments and Optoelectronics Engineering, Tianjin University, Tianjin, China; ^3^Tianjin Tianshi College, Tianjin, China; ^4^Department of Computer and Network Engineering, College of Information Technology, United Arab Emirates University, Al Ain, United Arab Emirates

**Keywords:** brain-computer interface, EEG, fNIRS, stroke, balance rehabilitation

## Abstract

Balance rehabilitation is exceedingly crucial during stroke rehabilitation and is highly related to the stroke patients’ secondary injuries (caused by falling). Stroke patients focus on walking ability rehabilitation during the early stage. Ankle dorsiflexion can activate the brain areas of stroke patients, similar to walking. The combination of electroencephalography (EEG) and functional near-infrared spectroscopy (fNIRS) was a new method, providing more beneficial information. We extracted the event-related desynchronization (ERD), oxygenated hemoglobin (HBO), and Phase Synchronization Index (PSI) features during ankle dorsiflexion from EEG and fNIRS. Moreover, we established a linear regression model to predict Berg Balance Scale (BBS) values and used an eightfold cross validation to test the model. The results showed that ERD, HBO, PSI, and age were critical biomarkers in predicting BBS. ERD and HBO during ankle dorsiflexion and age were promising biomarkers for stroke motor recovery.

## Introduction

Stroke is a disease affecting the arteries within the brain, resulting in motor impairment in about 80% of survivors (Langhorne et al., 2009). Among many stroke survivors, most patients were left with sequelae of motor dysfunction, and 30% of patients completely lost the ability to work and became highly disabled (Langhorne et al., 2009; Benjamin et al., 2017). Motor dysfunction causes patients to lose part of their living ability, rendering them unable to complete some daily living activities (Basteris et al., 2014). Therefore, motor recovery always focuses on stroke rehabilitation (Hatem et al., 2016). Balance recovery is essential to motor recovery, as the imbalance-leading falling substantially affects regular training and rehabilitation. In clinical practice, the Berg Balance Scale (BBS) is often used to evaluate the balance function of patients with cerebrovascular and brain injury (Sapmaz and Mujdeci, 2021). However, the scale’s accuracy depends on the experience and subjective judgment of the physical therapists. A biomarker that can illustrate the balance recovery process is necessary to organize the rehabilitation strategy better and improve balance recovery. Developed imaging techniques have given valuable information for diagnostic and functional prognosis. Nevertheless, they may have limitations, such as the special requirements for patients and low temporal resolution (Mukherjee et al., 2008; Buchbinder, 2016). Therefore, more and more studies have concentrated on more convenient methods with electroencephalography (EEG) (Wu et al., 2016; Sebastian-Romagosa et al., 2020).

The EEG acquisition device is simple and portable and has a high temporal resolution. It is highly sensitive to detecting EEG activities and allows subjects to perform some complex limb movement tasks while observing them non-invasively and dynamically in real-time. The neurons’ activity in the brain has been broadly used to monitor the stroke survivors’ brain states (Cillessen et al., 1994; Foreman and Claassen, 2012; Xin et al., 2017). The EEG’s beta band power patterns differed according to the location of the lesion (Park et al., 2016), and event-related desynchronization (ERD) magnitude correlated with residual motor function in the paretic arm (Bartur et al., 2019). However, one challenge of using EEG is its low spatial resolution problem, i.e., the ERD may be contaminated and weakened by the neural activities in the nearby areas. One alternative solution is to use functional near-infrared spectroscopy (fNIRS) as a supplement (Li et al., 2020). In a study using fNIRS to assess the correlation between cortical activation and external postural disturbances, the correlation became stronger with an increase in position-related oxygenated hemoglobin signal and an increase in balance function as measured by the BBS balance scale supplementary motor area (SMA) (Fujimoto et al., 2014). The fNIRS alone has been applied to assess the stroke’s progressive brain plasticity (Delorme et al., 2019). It has also been used with EEG to estimate the effect of different training strategies (Wang et al., 2019). Therefore, combining fNIRS and EEG may give new sight to stroke rehabilitation assessment.

The stroke rehabilitation assessment with EEG or fNIRS was usually undertaken during resting tasks (Nicolo et al., 2015; Sebastian-Romagosa et al., 2020). However, motor recovery should be reflected better during motor or motor imagery tasks (Wang et al., 2019; Li et al., 2020) when the corresponding brain area is activated. Walking ability is an urgent need for stroke patients in the early stage. The assessment should be taken during walking to assess the walking ability of stroke patients precisely. Bipedal locomotion is a complex task requiring maintaining specific motion frequencies, balance and load-bearing, visual integration, and multi-joint coordination (Petersen et al., 2012). However, most stroke survivors during the early stage cannot walk, or they may fall off during walking.

Additionally, ankle dorsiflexion is critical for walking as it occurs throughout the swing phase and at the initiation of the stance phase of gait (Dobkin et al., 2004). How the stroke survivors complete the ankle dorsiflexion affects their walking ability. Therefore, ankle dorsiflexion may be a promising task for stroke rehabilitation assessment (Gennaro and De Bruin, 2020).

This paper aims to evaluate the combination of EEG and fNIRS features during ankle dorsiflexion in rehabilitation assessment. We collected data from stroke survivors during ankle dorsiflexion and built a linear regression model with age, ERD, and oxygenated hemoglobin (HBO) as the predictors and BBS as the response. Our results verified the feasibility of EEG and fNIRS combination in predicting stroke balance state.

## Materials and methods

### Participants and experiments

Eight participants (three females and five males; mean age: 53.5 ± 15.48 years old) with stroke participated. All participants suffered hemiplegia from the first unilateral stroke, resulting in lower limb function limitation without sensory function loss. They needed to understand written and oral instructions and be in a good mental state, with a mini-mental state examination (MMSE) score > 24. The healthy control group consisted of six age-matched adults. All participants gave their written informed consent prior to participation. The Ethical Committee of Tianjin Medical University General Hospital approved the study.

**FIGURE 1:**
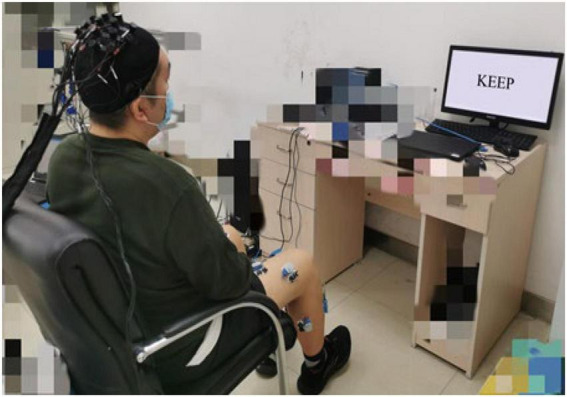
Experimental scenario.

Before the experiment, the motor function of the participant was assessed by three physical therapists with the BBS scale for lower extremities. The mean of the scale values was recorded. The participants were seated in a chair during the experiment, with their feet naturally on the ground. They were asked to complete the paraplegic dorsiflexion according to the instructions on the screen as in Figure 1.

**FIGURE 2:**
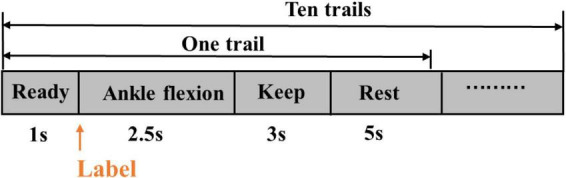
A session trail.

During the experiment, there were five sessions, each including ten trials. A single trial lasted for 11.5 s. Thus, it consisted of 1-s preparation, 2.5-s dynamic dorsiflexion, three-second static dorsiflexion maintenance, and five-second rest (Figure 2). A 5-min rest between successive sessions in case of muscle fatigue existed.

The participants were asked to relax their upper body, keep their upper body and head as still as possible, and avoid moving their heads, talking, swallowing, and blinking excessively during the dorsiflexion. The stimulation interface was completed based on E-Prime 3.0 software (Psychology Software Tools, Pittsburgh, PA, United States).

### Data recording

**FIGURE 3:**
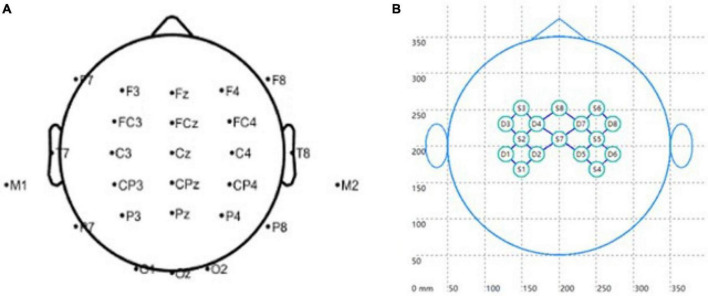
Signal acquisition. **(A)** EEG electrodes, and **(B)** fNIRS probe arrangement.

EEG signals were recorded by the Neuroscan Greal EEG system (Neuroscan, Victoria, Australia). Twenty-eight-lead EEG signals were collected according to the 10/20 system, as in Figure 3A. The sampling rate of the EEG signal was 1,024 Hz, with the top of the head as a reference. The ground electrode was placed on the GND of the forehead.

## Data processing

### ERD

As ankle flexion was highly related to the activities of neurons under Cz, the collected raw EEG signals at Cz were pre-processed. Data pre-processing was performed using Matlab R2014b (Math Works, MA, United States) with the toolbox EEGLAB (Swartz Center for Computational Neuroscience^1^). EEG signals were filtered to 0.05–35 Hz, and the EEG was down-sampled to 256 Hz. Then, eye movements and excessive muscle activity components were identified by visual inspection and removed after independent component analysis (ICA). Data were intercepted from 1 s before to 5 s after the onset of ankle dorsiflexion (0 s), and −1 s to 0 s was the baseline.

The event-related power changes can be shown in the time-frequency domain by event-related spectral perturbation (ERSP), an excellent method to evaluate the time-frequency characteristics of event-related potentials. ERSP could provide information for event-related synchronization (ERS) and event-related desynchronization (ERD). It considers the average power spectrum changes of event-related potentials in a frequency band range. The ERSP for n-trial data was calculated as:


(1) $$E\,R\,S\,P\,\left(f,t\right)=\frac{1}{n}\,\sum_{k=1}^{n}F_{k}\,{\left(f,t\right)}^{2}$$

where *n* indicated the total trial number, and *F*_*k*_(*f*,*t*) the spectral estimation at frequency *f* and time *t* for the *k*th trial.

To show ERD/ERS during the task, we calculated the baseline-normalized ERSP. For each time bin, the normalized ERSP across frequency *f* was obtained by


(2) n⁢E⁢R⁢S⁢P⁢(f)=E⁢R⁢S⁢P⁢(f)-b⁢a⁢s⁢e⁢l⁢i⁢n⁢e⁢(f)

where $b\,a\,s\,e\,l\,i\,n\,e\,\left(f\right)=\frac{1}{N}\,\sum_{t=-1\,s}^{0\,s}E\,R\,S\,P\,\left(f,t\right)$, *N* was the number of time bins from −1 s to 0 s. The ERD index, the predictor of the BBS scale value, was the mean of negative nERSP within a specific area (15 Hz∼23 Hz and 0 s∼1 s), determined by the average of nERSP in the time-frequency domain.

### Phase synchronization index

The brain’s function depends on the interaction between neurons in different regions or across brain regions (He et al., 2007). Recent studies have also demonstrated the efficacy of synchronized brain activity in evaluating neural networks and their relationship with various clinical conditions (Engel et al., 2013). Brain damage after a stroke can change brain function connections. Therefore, the multi-regional interactions in the brain network are valuable for understanding the pathophysiology and neurological dysfunction after a stroke (Du et al., 2018).

The phase synchronization index is a normalized parameter that measures the relationship between a pair of variables and effectively describes the integration between neurons. First, the pre-processed signal is filtered to the band of interest, and then the instantaneous phase of the filtered EEG signal is extracted using the Hilbert transform. The phase synchronization index is calculated as follows, quantifying the phase synchronization level of the signal:


(3) $$P\,S\,I=\sqrt{{\left\langle \cos\,\phi_{x\,y}^{H}\left(t\right)\right\rangle }_{t}^{2}+{\left\langle \sin\,\phi_{x\,y}^{H}\left(t\right)\right\rangle }_{t}^{2}}$$

which, $\phi_{x\,y}^{H}\left(t\right)$ is the instantaneous phase difference between the signals *x*(*t*) and *y*(*t*), and ⟨.⟩ represents the mean operation over some time. PSI is a real number between 0 and 1. When PSI = 1, the two signals are completely synchronized, and when PSI = 0, they are entirely out of sync.

A multi-channel oxygenation monitor (NirScan, Danyang Huichuang Medical Equipment Co., Ltd.) recorded the participants’ hemodynamic responses over sensorimotor cortex areas. The distance between the source and the detector was 3 cm, and the sampling frequency was 20 Hz. The layout of the fNIRS acquisition cap is in Figure 3B, where S1-S8 represents fNIRS emission source probes and D1-D8 fNIRS detector probes. Thus, there were twenty-two fNIRS channels in total.

### Oxygenated, deoxygenated, and total hemoglobin

The oxygenation monitor software calculated the oxygenated, deoxygenated, and total hemoglobin (HBO, HBR, and HBT). Then the data were 0.01 Hz∼0.2 Hz filtered to exclude disturbance. The data within the area covered by S2-D4, S2-D2, S7-D2, S7-D4, S7-D7, S7-D5, S5-D7, and S5-D5 were segmented from 2 s before (baseline) to 10 s after ankle flexion. The task-related changes of HBO, HBR, and HBT according to the 2-s baseline were calculated and averaged for the above fNIRS channels. The area between the average HBO curve and the horizontal axis predicted the BBS scale values.

### Linear regression method

Linear regression analysis is a common modeling method; its fundamental principle is to use one or more independent variables to predict a dependent variable to establish a linear relationship. Assuming that the response variable Y is a linear function of one or more predictive variables (explanatory variables) X, multiple linear regression can be expressed as:


(4) $$Y=\beta_{0}+\beta_{1}\,X_{1}+\beta_{2}\,X_{2}+\cdots +\beta_{n}\,X_{n}+e$$

where *Y* is the target variable, β_*i*_ (*i* = 0, 1, 2. *n*) is the regression parameters, *X*_*i*_ = (*i* = 0, 1, 2. *n*) is the predictive variable of regression. The *e* is the error term.

After building the model, it is usually necessary to analyze whether the linear influence of the predictive variable *X_i_* on the target variable *Y* is significant further. Next, we select the predictive variable with the most significant effect on the target variable *Y* and then determine the relative weight of each independent variable on the target variable.

This paper explored the correlation between BBS and multiple variables, including age, ERD, HBO and the square of HBO. A 4 × 8 variable existed for linear regression. The coefficient of determination, R^2^, was used to evaluate the goodness of fit and statistical significance for each predictor with a pre-set alpha level of 0.05 for analysis. In order to further demonstrate and evaluate the effectiveness of the model, an eightfold cross validation of the model was done, with seven samples as the data for model establishment, and one sample for testing. The RMSE was calculated for the cross validation.

## Results

### ERD features

**FIGURE 4:**
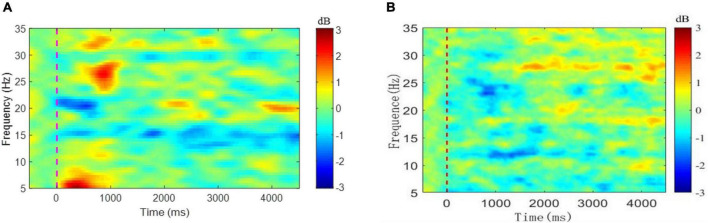
Mean of nERSP at Cz for **(A)** 8 patients and **(B)** 6 healthy control group.

Figure 4A depicts the mean nERSP at Cz in a total of 400 tests of ankle flexion in eight stroke patients, while Figure 4B illustrates the mean nERSP at Cz in a total of 300 tests of ankle flexion in six healthy controls. Blue represents ERD and red ERS region. The ankle enters a dynamic buckling process within 2.5 s after 0 s and a static contraction after 2.5 s considering −1 s∼0 s as the baseline. The figure reveals that the patient had an apparent ERD phenomenon at about 15 Hz (lasting the entire exercise cycle) and about 20 Hz (mainly during dynamic dorsiflexion within 1 s after the start of the movement and declining during the rest period). However, the evident ERD phenomenon of healthy subjects appeared at about 13 Hz and 25 Hz, and later than patients, and lasted shorter in the whole movement cycle. ERD phenomenon showed that cortical regions of the Cz electrode are convoluted in task planning and motion control during the task process, aligning with previous studies (Xu et al., 2018). Results verified that the paralyzed ankle flexion induced ERD within the beta band at Cz.

**FIGURE 5:**
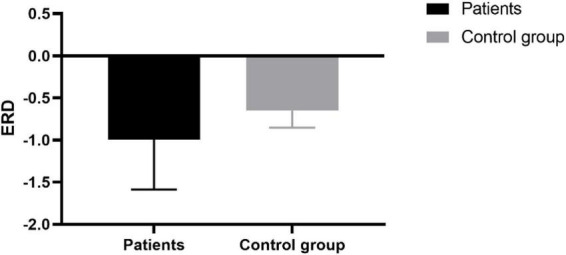
ERD index of patients and the control group.

This study used the ERD index as the predictor of the BBS scale, the mean value of negative nERSP within a specific area (15 Hz∼23 Hz and 0 s∼1 s) determined by the mean nERSP in the time-frequency domain. In Figure 5, the calculated mean ERD of 8 patients was higher than that of the healthy controls. However, no significant difference between the two, possibly due to the limited sample size.

#### Phase synchronization index

**FIGURE 6:**
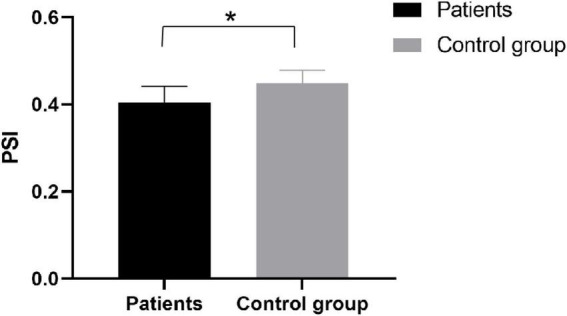
PSI index of patients and the control group. *Significant difference.

We compared the phase synchronization index of EEG signals. We found significant differences (*p* = 0.023) in inter-PSI between patients and healthy subjects at the α band (Figure 6), a better understanding the differences between patients and healthy subjects during exercise. It suggested the stroke inactivation of the cortical network. The difference in PSI between hemispheres also indicated that the dynamic mode of synchronous activation between the two hemispheres deteriorated in functional communication due to the brain injury.

### HBO features

**FIGURE 7:**
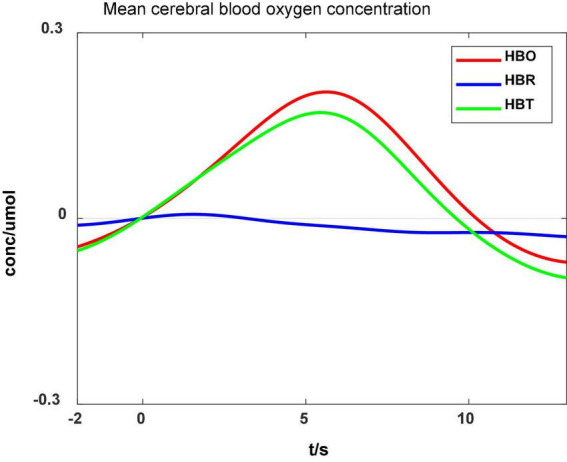
Cerebral blood oxygen concentration during ankle flexion.

Figure 7 shows that the mean HBO, HBR, and HBT values varied during ankle flexion: the HBO and HBT increased slowly after the ankle flexion onset (0 s) and decreased after the flexion completion (5.5 s). The fNIRS data differed consistently with ankle flexion, but the change lasted longer than the movement. HBO closely relates to the metabolism of local tissues. Therefore, the measured HBO can reflect the motor area activities.

### Regression analysis

Table 1 lists the peak of the HBO curve for every patient. Then, this index should reflect the oxygen consumption of the specific brain area. It also depicts the ERD indexes at Cz of eight participants and the corresponding average values. We used the values in this table for the regression analysis.

**TABLE 1:** Age, ERD, HBO, and BBS of the patients.

Patient	Age	ERD	HBO	BBS
P1	60	−1.834	1.905	55
P2	74	−0.396	1.999	33
P3	49	−1.977	0.211	40
P4	64	−0.695	0.098	8
P5	33	−0.979	2.925	46
P6	32	−0.448	2.066	55
P7	67	−0.825	1.170	40
P8	49	−0.790	2.000	40
Avg.	54	−0.993	1.547	39.6

The multiple linear regression model was established using the data in Table 1. Table 2 lists the beta coefficient and p- values for all the model parameters. The adjusted R^2^ was 0.840, indicating the model explained 84% of the variability in BBS. Correlation analysis revealed that no significant correlation between any two of the three predictors existed.

**TABLE 2:** Multiple linear regression model.

Independent variable	β (95% CL)	*p* value
Constant	30.6 (17.2, 44.0)	0.107
ERD	−12.4 (−16.5, −8.4)	0.054
HBO	37.3 (28.9, 45.8)	0.021
AGE	−0.5 (−0.7, −0.3)	0.075
HBO*HBO	−10.6 (−13.7, −7.4)	0.043

*Multiplication sign.

**FIGURE 8:**
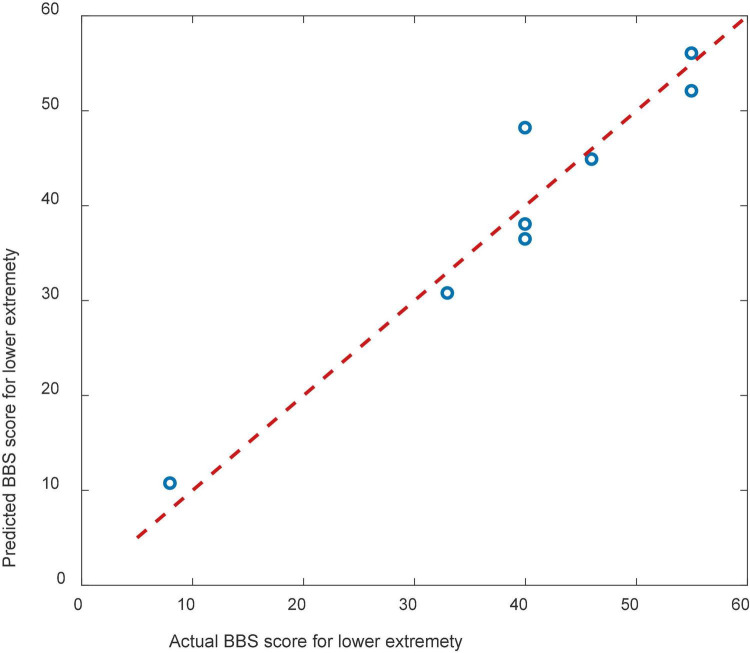
Scatterplots showing the relations between the actual and predicted BBS scales.

The regression model showed that the predictors ERD, AGE, and HBO could predict patients’ balance function (Figure 8), and the prediction model was significant (*p* = 0.043).

**FIGURE 9:**
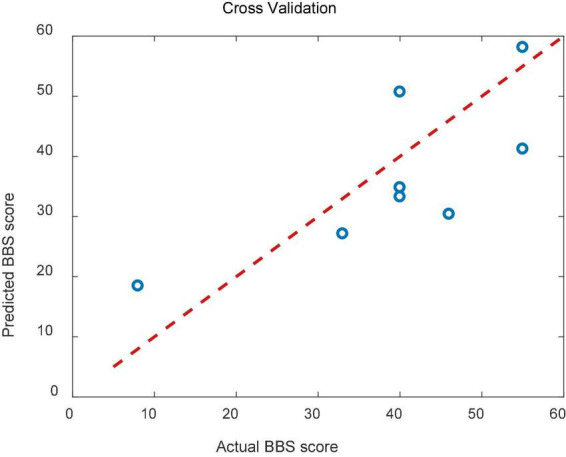
Scatterplots showing the relations between the actual and predicted BBS scales for cross validation.

The result of cross validation was shown in Figure 9. The RMSE of the predicted and actual BBS scores was 9.83 for the cross validation.

## Discussion and conclusion

We focused on event-related desynchronization (ERD) as a motor command. ERD is a phenomenon where the α and/or the β band voltages decrease as the number of synchronized neural assemblies increases. Theα and β ERD occur before and during motor execution or motor imagery (Babiloni et al., 1999). Therefore, ERD can be interpreted as an electrophysiological correlate of activated cortical areas involved in processing sensory or cognitive information or the production of motor behavior (Pfurtscheller, 1992). An increased and/or more widespread ERD could result from the involvement of a more extensive neural network or more cell assemblies in information processing. Factors contributing to such an enhancement of the ERD include increased task complexity and more efficient task performance (Defebvre et al., 1996). This study covers dorsiflexion of the ankle joint as simple and easy for healthy subjects. However, they require more attention and energy for stroke patients due to motor dysfunction. It may also explain why ERD is more pronounced in patients than in healthy subjects. The ERD of healthy people during dynamic contraction of the ankle joint is more noticeable than that during static contraction, which may be due to activity in primary sensorimotor areas increasing in association with learning a new motor task and decreasing after the task has been learned (Zhuang et al., 1997).

In the fNIRS study, when subjects performed the ankle flexion task, the sensorimotor area of the cerebral cortex consumed oxygen and energy. At this point, the over-compensation mechanism of the brain blood supply system would flood the region with oxygen-rich blood, increasing HBO concentration and decreasing HBR. It further suggests that the experimental task activated this brain region. Previous studies have demonstrated that EEG and fNIRS-based brain-computer interface technology can enable a broader range of cortical activation in stroke patients, enhancing neuroplasticity (Kaiser et al., 2014). In this study, EEG features, including ERD, fNIRS, and HBO, can predict patients’ balance function, providing a new idea for guiding the rehabilitation of stroke patients and evaluating and predicting patients’ rehabilitation status. We considered the mean ERD and HBO values in this study. However, more features should be extracted from the data providing varied information. Future studies should address these features. Only eight stroke patients and six healthy control groups were recruited for our experiment, providing a relatively small number of subjects. The result of the regression analysis should be further validated after collecting more data from patients and healthy control.

To sum up, it was the first study considering both EEG and fNIRS features during ankle dorsiflexion as the biomarkers for stroke assessment. We extracted ERD and HBO features from eight stroke patients and established a linear regression model to predict BBS scale values. Age, ERD, and HBO during ankle dorsiflexion were promising biomarkers for stroke motor recovery. Further studies should include more participants with stroke and healthy controls to obtain a reliable relationship between these features and motor function state.

## Data availability statement

The original contributions presented in this study are included in the article/supplementary material, further inquiries can be directed to the corresponding author/s.

## Ethics statement

The studies involving human participants were reviewed and approved by Ethics Committee of Tianjin Medical University General Hospital. The patients/participants provided their written informed consent to participate in this study.

## Author contributions

JL and FL completed the ethics files of this experiment. JL, YS, and SL recorded the original experiment data, analyzed the experiment data, and penned the manuscript. YW and AB wrote parts of the manuscript. CW designed the experiment and revised the manuscript. All authors contributed to the article and approved the submitted version.

## Footnotes

1
http://sccn.ucsd.edu/eeglab/

## References

[B1] Babiloni C. Carducci F. Cincotti F. Rossini P. M. Neuper C. Pfurtscheller G. (1999). Human movement-related potentials vs desynchronization of EEG alpha rhythm: A high-resolution EEG study. *Neuroimage* 10 658–665. 10.1006/nimg.1999.0504 10600411

[B2] Bartur G. Pratt H. Soroker N. (2019). Changes in mu and beta amplitude of the EEG during upper limb movement correlate with motor impairment and structural damage in subacute stroke. *Clin. Neurophysiol.* 130 1644–1651. 10.1016/j.clinph.2019.06.008 31326646

[B3] Basteris A. Nijenhuis S. M. Stienen A. H. A. Buurke J. H. Prange G. B. Amirabdollahian F. (2014). Training modalities in robot-mediated upper limb rehabilitation in stroke: A framework for classification based on a systematic review. *J. Neuroeng. Rehabil.* 11:111. 10.1186/1743-0003-11-111 25012864 PMC4108977

[B4] Benjamin E. J. Blaha M. J. Chiuve S. E. Cushman M. Das S. R. Deo R. (2017). Heart disease and stroke statistics-2017 update a report from the American heart association. *Circulation* 135 E146–E603. 28122885 10.1161/CIR.0000000000000485 PMC5408160

[B5] Buchbinder B. R. (2016). Functional magnetic resonance imaging. *Handb. Clin. Neurol.* 135 61–92. 27432660 10.1016/B978-0-444-53485-9.00004-0

[B6] Cillessen J. P. Van Huffelen A. C. Kappelle L. J. Algra A. van Gijn J. (1994). Electroencephalography improves the prediction of functional outcome in the acute stage of cerebral ischemia. *Stroke* 25 1968–1972. 8091439 10.1161/01.str.25.10.1968

[B7] Defebvre L. Bourriez J. L. Destee A. Guieu J. D. (1996). Movement related desynchronisation pattern preceding voluntary movement in untreated Parkinson’s disease. *J. Neurol. Neurosurg. Psychiatry* 60 307–312. 10.1136/jnnp.60.3.307 8609509 PMC1073855

[B8] Delorme M. Vergotte G. Perrey S. Froger J. Laffont I. (2019). Time course of sensorimotor cortex reorganization during upper extremity task accompanying motor recovery early after stroke: An fNIRS study. *Restor. Neurol. Neurosci.* 37 207–218. 10.3233/RNN-180877 31227675

[B9] Dobkin B. H. Firestine A. West M. Saremi K. Woods R. (2004). Ankle dorsiflexion as an fMRI paradigm to assay motor control for walking during rehabilitation. *Neuroimage* 23 370–381. 10.1016/j.neuroimage.2004.06.008 15325385 PMC4164211

[B10] Du J. Hu J. Hu J. Xu Q. Zhang Q. Liu L. (2018). Aberrances of cortex excitability and connectivity underlying motor deficit in acute stroke. *Neural Plast.* 2018:1318093. 10.1155/2018/1318093 30420876 PMC6215555

[B11] Engel A. K. Gerloff C. Hilgetag C. C. Nolte G. (2013). Intrinsic coupling modes: Multiscale interactions in ongoing brain activity. *Neuron* 80 867–886. 24267648 10.1016/j.neuron.2013.09.038

[B12] Foreman B. Claassen J. (2012). Quantitative EEG for the detection of brain ischemia. *Crit. Care* 16:216. 10.1186/cc11230 PMC3681361 22429809

[B13] Fujimoto H. Mihara M. Hattori N. Hatakenaka M. Kawano T. Yagura H. (2014). Cortical changes underlying balance recovery in patients with hemiplegic stroke. *Neuroimage* 85 547–554. 10.1016/j.neuroimage.2013.05.014 23684871

[B14] Gennaro F. De Bruin E. D. (2020). A pilot study assessing reliability and age-related differences in corticomuscular and intramuscular coherence in ankle dorsiflexors during walking. *Physiol. Rep.* 8:e14378. 10.14814/phy2.14378 32109345 PMC7048377

[B15] Hatem S. M. Saussez G. Della Faille M. Prist V. Zhang X. Dispa D. (2016). Rehabilitation of motor function after stroke: A multiple systematic review focused on techniques to stimulate upper extremity recovery. *Front. Hum. Neurosci.* 10:442. 10.3389/fnhum.2016.00442 27679565 PMC5020059

[B16] He B. J. Snyder A. Z. Vincent J. L. Epstein A. Shulman G. L. Corbetta M. (2007). Breakdown of functional connectivity in frontoparietal networks underlies behavioral deficits in spatial neglect. *Neuron* 53 905–918. 17359924 10.1016/j.neuron.2007.02.013

[B17] Kaiser V. Bauernfeind G. Kreilinger A. Kaufmann T. Kübler A. Neuper C. (2014). Cortical effects of user training in a motor imagery based brain-computer interface measured by fNIRS and EEG. *Neuroimage* 85 432–444. 10.1016/j.neuroimage.2013.04.097 23651839

[B18] Langhorne P. Coupar F. Pollock A. (2009). Motor recovery after stroke: A systematic review. *Lancet Neurol.* 8 741–754. 19608100 10.1016/S1474-4422(09)70150-4

[B19] Li R. Li S. Roh J. Wang C. Zhang Y. (2020). Multimodal neuroimaging using concurrent EEG/fNIRS for poststroke recovery assessment: An exploratory study. *Neurorehabil. Neural Repair* 34 1099–1110. 10.1177/1545968320969937 33190571

[B20] Mukherjee P. Berman J. I. Chung S. W. Hess C. P. Henry R. G. (2008). Diffusion tensor MR imaging and fiber tractography: Theoretic underpinnings. *Am. J. Neuroradiol.* 29 632–641. 18339720 10.3174/ajnr.A1051 PMC7978191

[B21] Nicolo P. Rizk S. Magnin C. Pietro M. D. Schnider A. Guggisberg A. G. (2015). Coherent neural oscillations predict future motor and language improvement after stroke. *Brain* 138 3048–3060. 10.1093/brain/awv200 26163304

[B22] Park W. Kwon G. H. Kim Y.-H. Lee J. H. Kim L. (2016). EEG response varies with lesion location in patients with chronic stroke. *J. Neuroeng Rehabil.* 13:21. 10.1186/s12984-016-0120-2 26935230 PMC4776402

[B23] Petersen T. H. Willerslev-Olsen M. Conway B. A. Nielsen J. B. (2012). The motor cortex drives the muscles during walking in human subjects. *J. Physiol.* 590 2443–2452. 22393252 10.1113/jphysiol.2012.227397 PMC3424763

[B24] Pfurtscheller G. (1992). Event-related synchronization (ERS): An electrophysiological correlate of cortical areas at rest. *Electroencephalogr. Clin. Neurophysiol.* 83 62–69. 10.1016/0013-4694(92)90133-3 1376667

[B25] Sapmaz M. Mujdeci B. (2021). The effect of fear of falling on balance and dual task performance in the elderly. *Exp. Gerontol.* 147:111250. 10.1016/j.exger.2021.111250 33493582

[B26] Sebastian-Romagosa M. Udina E. Ortner R. Dinarès-Ferran J. Cho W. Murovec N. (2020). EEG biomarkers related with the functional state of stroke patients. *Front. Neurosci.* 14:582. 10.3389/fnins.2020.00582 32733182 PMC7358582

[B27] Wang Z. Zhou Y. Chen L. Gu B. Yi W. Liu S. (2019). BCI monitor enhances electroencephalographic and cerebral hemodynamic activations during motor training. *IEEE Trans. Neural. Syst. Rehabil. Eng.* 27 780–787. 30843846 10.1109/TNSRE.2019.2903685

[B28] Wu J. Srinivasan R. Quinlan E. B. Solodkin A. Small S. L. Cramer S. C. (2016). Utility of EEG measures of brain function in patients with acute stroke. *J. Neurophysiol.* 115 2399–2405. 26936984 10.1152/jn.00978.2015 PMC4922461

[B29] Xin X. Chang J. Gao Y. Shi Y. (2017). Correlation between the revised brain symmetry index, an eeg feature index, and short-term prognosis in acute ischemic stroke. *J. Clin. Neurophysiol.* 34 162–167. 10.1097/WNP.0000000000000341 27584547

[B30] Xu R. Wang Y. Wang K. Zhang S. He C. Ming D. (2018). Increased corticomuscular coherence and brain activation immediately after short-term neuromuscular electrical stimulation. *Front. Neurol.* 9:886. 10.3389/fneur.2018.00886 30405518 PMC6206169

[B31] Zhuang P. Toro C. Grafman J. Manganotti P. Leocani L. Hallett M. (1997). Event-related desynchronization (ERD) in the alpha frequency during development of implicit and explicit learning. *Electroencephalogr. Clin. Neurophysiol.* 102 374–381. 10.1016/s0013-4694(96)96030-7 9146500

